# Similarities in Gene Expression Profiles during *In Vitro* Aging of Primary Human Embryonic Lung and Foreskin Fibroblasts

**DOI:** 10.1155/2015/731938

**Published:** 2015-08-03

**Authors:** Shiva Marthandan, Steffen Priebe, Mario Baumgart, Marco Groth, Alessandro Cellerino, Reinhard Guthke, Peter Hemmerich, Stephan Diekmann

**Affiliations:** ^1^Leibniz-Institute for Age Research-Fritz Lipmann Institute e.V. (FLI), 07745 Jena, Germany; ^2^Leibniz Institute for Natural Product Research and Infection Biology-Hans-Knöll-Institute e.V. (HKI), 07745 Jena, Germany; ^3^Laboratory of Neurobiology, Scuola Normale Superiore, University of Pisa, 56126 Pisa, Italy

## Abstract

Replicative senescence is of fundamental importance for the process of cellular aging, since it is a property of most of our somatic cells. Here, we elucidated this process by comparing gene expression changes, measured by RNA-seq, in fibroblasts originating from two different tissues, embryonic lung (MRC-5) and foreskin (HFF), at five different time points during their transition into senescence. Although the expression patterns of both fibroblast cell lines can be clearly distinguished, the similar differential expression of an ensemble of genes was found to correlate well with their transition into senescence, with only a minority of genes being cell line specific. Clustering-based approaches further revealed common signatures between the cell lines. Investigation of the mRNA expression levels at various time points during the lifespan of either of the fibroblasts resulted in a number of monotonically up- and downregulated genes which clearly showed a novel strong link to aging and senescence related processes which might be functional. In terms of expression profiles of differentially expressed genes with age, common genes identified here have the potential to rule the transition into senescence of embryonic lung and foreskin fibroblasts irrespective of their different cellular origin.

## 1. Introduction

Cellular senescence is a terminal phase observed towards the end of a primary human fibroblast cell population after numerous cell divisions; it is considered to be the cellular aging process. Cellular senescence occurs either naturally or stress induced; that is, cells stop dividing after a finite number of cell divisions (termed “replicative senescence”), reaching the final cell cycle arrested state called the “Hayflick limit” [1]. The process of senescence is associated with a number of phenotypes; in general, the integrity and function of tissues decline, resulting in the body being susceptible to diseases associated with age [2, 3]. Key factors driving cellular senescence are induced increase in Cyclin dependent kinase inhibitors (CDKIs) [4], oxidative stress [5], and DNA damage [6, 7]. In senescence, despite their viability and active metabolism, cells are resistant to mitogenic or apoptotic stimuli [8, 9]. On the one hand, cellular senescence results in irreversible growth arrest, limiting the proliferation of damaged cells susceptible to neoplastic transformation resulting in a decreased incidence of cancer. However, on the other hand, senescence results in* in vivo* aging, weakening the function and renewal of stem cells [10]. Markers are able to identify cellular senescence* in vitro* and* in vivo*: enlarged cell morphology, increase in amount of cellular debris, changes in chromatin structure, increase in Cyclin dependent kinase inhibitors (CDKIs) expression, presence of senescence associated secretory phenotype (SASP), and senescence associated ß-galactosidase (SA ß-Gal) [11–13]. DNA damage response and the p53-p21 and p16-pRb pathways are crucial for senescence induction [14], together with additional pathways including telomere uncapping, DNA damage (UV, ionizing radiation, and chemicals), cytoskeletal genes, the interferon pathway, nutrient imbalances, oncogenic activities, and oxidative stress [7, 15, 16]. In primates, the percentage of senescent skin fibroblasts increases with age* in vivo* [17]. Here, we therefore used primary human fibroblasts [9, 13, 18] as our model system.

Recently, we identified individual gene expression patterns during replicative senescence among five fibroblast cell lines of different cell origins [13]. In this study, we determined mRNA expression changes during different stages of their lifespan in two fibroblast cell lines of different cell origin. We analyzed the transcriptome, determined by RNA-seq, at five separate population doublings (PDs) between young and senescent embryonic lung (MRC-5) and foreskin (HFF) fibroblasts. Using both molecular and systems biology approach, we studied the growth pattern of the two fibroblast cell lines in detail. By comparing fibroblasts from two different origins we were able to determine either mRNA changes specific for one of the cell lines or common transcriptomic patterns which underlie the process of replicative senescence.

## 2. Materials and Methods

### 2.1. Cell Lines

Primary human fibroblasts (MRC-5, primary cells, 14-week-gestation male, fibroblasts from normal lung, normal diploid karyotype) were obtained from ATCC (LGC Standards GmbH, Wesel, Germany). Human foreskin fibroblasts (HFFs; primary cells, fibroblasts from foreskin, normal diploid karyotype) cells were kind gifts of T. Stamminger (University of Erlangen [19]).

### 2.2. Cell Culture

Primary human fibroblast cells were cultured in Dulbeccos Modified Eagle's Low Glucose Medium (DMEM) with L-glutamine (PAA Laboratories, Pasching, Austria), supplemented with 10% fetal bovine serum (FBS) (PAA Laboratories) under normal air conditions in a 9.5% CO_2_ atmosphere at 37°C. The cells were subcultured by removing the remaining medium followed by washing in 1x PBS (pH 7.4) (PAA Laboratories) and detachment using trypsin/EDTA (PAA Laboratories). Primary fibroblasts were subcultured in a 1 : 4 (= 2 population doublings (PDs)) or 1 : 2 (= 1 PD) ratio. For stock purposes, cryoconservation of the cell lines at various PDs was undertaken in cryoconserving medium (DMEM + 10% FBS + 5% DMSO). Cells were immediately frozen at −80°C and stored for two to three days. Afterwards, cells were transferred to liquid nitrogen for long time storage. Refreezing and rethawing was not performed to avoid premature senescence [20].

A vial of each of the two fibroblast cell lines (MRC-5 and HFF) was obtained and maintained in culture from an early PD. On obtaining enough stock on confluent growth of the fibroblasts in 75 cm^2^ flasks, cells were subcultured into three separate 75 cm^2^ flasks and were passaged until they were senescent in culture. At five different time points of the fibroblast's span in culture (MRC-5 = PDs 32, 42, 52, 62, and 72 and HFFs = PDs 16, 26, 46, 64, and 74), the total RNA was extracted and used for high-throughput sequencing.

### 2.3. Detection of Senescence Associated ß-Galactosidase (SA ß-Gal)

The SA *β*-Gal assay was performed as described by [11] at each of the five PDs in both MRC-5 and HFF. Paired two-sample type 2 Student's *t*-tests assuming equal variances were done to examine the values obtained from SA ß-Gal assay for statistical significance [9].

### 2.4. Western Blotting

The protocol was carried out as explained in [9, 21]. The optimal concentration of all primary antibodies was estimated in primary human fibroblasts. Primary antibodies are as follows: anti-p21 mouse antibody (OP64; Calbiochem; dilution 1 : 200), anti-p15 rabbit antibody (4822; Cell Signaling Technology; 1 : 250), anti-p16 mouse antibody (550834; BD Pharmingen; 1 : 200), anti-p27 rabbit antibody (sc-528; Santa Cruz; 1 : 200), anti-Cyclin B1 mouse antibody (CCNB1; ab72; Abcam; 1 : 1000), anti-Eg5 rabbit antibody (KIF11; ab61199; Abcam; 1 : 500), anti-Histone H1.2 rabbit antibody (HIST1H1C; ab17677; Abcam; 1 : 1000), anti-ID3 mouse antibody (ab55269; Abcam; 1 : 100), anti-Cathepsin K rabbit antibody (CTSK; ab19027; Abcam; 1 : 50), anti-DKK3 goat antibody (ab2459; Abcam; 1 : 5000), anti-TMEM47 rabbit antibody (SAB1104840; SIGMA-Aldrich; 1 : 250), anti-IGFBP7 rabbit antibody (ab74169; Abcam; 1 : 500), anti-IGFBP2 rabbit antibody (ab91404; Abcam; 1 : 500), anti-MMP3 rabbit antibody (ab53015; Abcam; 1 : 200), anti-Thymosin beta 10 rabbit antibody (TMSB10; ab14338; Abcam; 1 : 10000), anti-Egr1 mouse antibody (ab55160; Abcam; 1 : 100), anti-RPS23 mouse antibody (ab57644; Abcam; 1 : 200), anti-LIF mouse antibody (SAB1406083; SIGMA-Aldrich; 1 : 100), anti-FBL rabbit antibody (SAB1101099; SIGMA-Aldrich; 1 : 500), anti-Id1 rabbit antibody (ab52998; Abcam; 1 : 500), anti-IL11 rabbit antibody (ab76589; Abcam; 1 : 500), anti-CLDN11 rabbit antibody (HPA013166; SIGMA-Life Sciences; 1 : 50), anti-NADH Dehydrogenase subunit 6 rabbit antibody (MT-ND6; ab81212; Abcam; 1 : 1000), anti-MT-ND5 rabbit antibody (ab83985; Abcam; 1 : 500), anti-Granulin rabbit antibody (GRN; ab108608; Abcam; 1 : 1000), anti-Cyclin D1 rabbit antibody (CCND1; 2922; Cell Signaling; 1 : 500), anti-Cyclin D2 mouse antibody (CCND2; ab3085; Abcam; 1 : 500), anti-Cyclin A2 rabbit antibody (CCNA2; NBP1-31330; Novus Biologicals; 1 : 1000), anti-Wnt16 rabbit antibody (ab109437; Abcam; 1 : 500), anti-Cystatin C rabbit antibody (CST3; ab109508; Abcam; 1 : 10000), anti-MOXD1 mouse antibody (SAB1409086; SIGMA-Aldrich; 1 : 200), anti-PERP rabbit antibody (ab5986; Abcam; 1 : 500) and anti-tubulin mouse antibody (T-9026; SIGMA-Aldrich; 1 : 5000). After development of film in the Western Blots procedure, intensity of the signals was quantified using Metamorph software [22]. The signal intensity values were examined for statistical significance using unpaired two-tailed two-sample Student's *t*-tests assuming unequal variances.

### 2.5. RNA Extraction

Total RNA was isolated using Qiazol (Qiagen) according to the manufacturer's protocol, with modifications as explained in [9].

### 2.6. Quantitative Real-Time PCR

Real-time PCR was performed using CFX384 thermocycler Biorad and Quantitect PCR system (Qiagen) as described earlier in [23]. Three reference genes (GAPDH, ACTB, and RAB10) were used for normalization of the CT values. Since our RNA-seq results revealed a stable expression of RAB10 for both cell lines across the PDs, it was selected as reference gene. An unpaired two-tailed two-sample Student's *t*-tests assuming unequal variances was used for examination for statistical significance based on the ΔCT values.

### 2.7. RNA Sequencing

For quality check, total RNA was analyzed using Agilent Bioanalyzer 2100 (Agilent Technologies) and RNA 6000 Nano Kit (Agilent) to ensure appropriate RNA quality in terms of degradation. The RNA integrity number (RIN) varies between 8 and 10 with an average of around 9.65. Total RNA was used for Illumina library preparation and next-generation sequencing [24]. About 2.5 *μ*g total RNA was used for indexed library preparation using Illumina's TruSeq RNA Sample Prep Kit v2 following the manufacturer's instruction. Libraries were pooled and sequenced (4 samples per lane) using a HiSeq2000 (Illumina) in single read mode with 50 cycles using sequencing chemistry v3. Sequencing resulted in approximately 43 million reads with a length of 50 bp (base pairs) per sample. Reads were extracted in FASTQ format using CASAVA v1.8.2 (Illumina).

### 2.8. RNA-seq Data Analysis

Raw sequencing data were received in FASTQ format. Read mapping was performed using Tophat 2.0.6 [25] and the human genome references assembly GRCh37.66 (http://feb2012.archive.ensembl.org). The resulting SAM alignment files were processed using featureCounts v1.4.3-p1 [26] and the respective GTF gene annotation, obtained from the Ensembl database [27]. Gene counts were further processed using the R programming language [28] and normalized to RPKM values. RPKM values were computed using exon lengths provided by featureCounts and the sum of all mapped reads per sample.

### 2.9. Sample Clustering and Analysis of Variance

**Figure 1:**
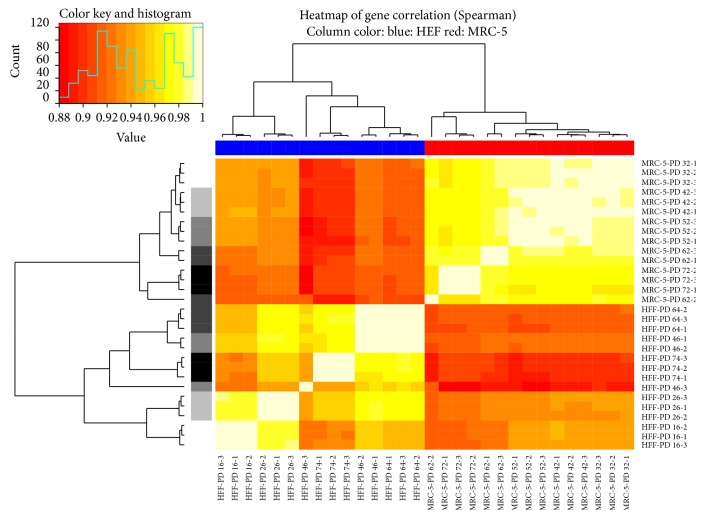
Heatmap showing Spearman correlation values computed for all expressed genes and all 30 samples sequences for this study. The histogram on top left denotes the distribution of the correlation values as well as the colors for each value. The dendrograms on top and left of the histogram are identical but show different colors at their leaves: top: color denotes the cell line (blue: HFF; red: MRC-5); left: color denotes the PD (bright to dark → young to old). Samples are clustered first according to cell line and second according to PD. Highest correlations were found between the three replicates (light areas on the main diagonal).

**Figure 2:**
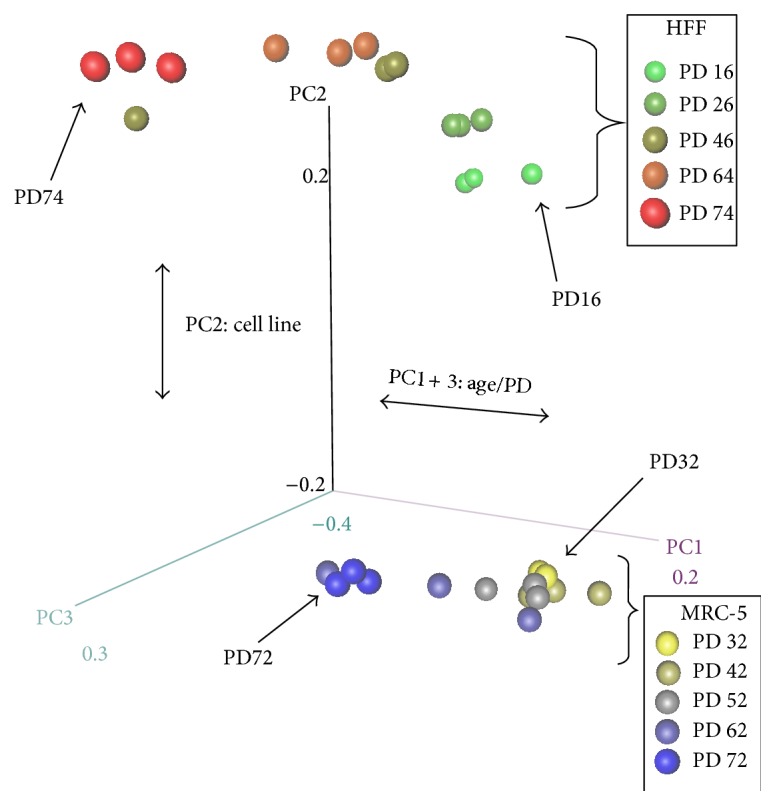
3D PCA plot for 30 samples (2 cell lines, 5 different PDs, triplicates). Both cell lines are clearly separated by PC2. The effect of aging is partly explained by PC1 and PC3. Colors: yellow to blue: young to old MRC-5 cells; green to red: young to old HFF cells.

Spearman correlation between all samples was computed in order to examine the variance and the relationship of global gene expression across the samples, using genes with raw counts larger than zero. Correlation values were visualized using a heatmap (Figure 1). Additionally, principal component analysis (PCA) was applied using the log 2 RPKM values for genes with raw counts larger than zero. Results were visualized in a three-dimensional scatterplot (Figure 2).

### 2.10. Detection of Differential Expression

The Bioconductor packages* DESeq* 1.10.4 [29] and* edgeR* 3.4.2 [30] were used to identify differentially expressed genes. Both packages provide statistics for determining of differential expression in digital gene expression data using a model based on the negative binomial distribution. The nonnormalized gene counts have been used here, since both packages include internal normalization procedures. The resulting *p* values were adjusted using Benjamini and Hochberg's approach for controlling the false discovery rate (FDR) [31]. Genes with an adjusted *p* value < 0.05 found by both packages were assigned as differentially expressed. Since large sets of DEG were found more strict selection cutoffs have been used: adjusted *p* value < 0.01 (by both packages) and absolute log 2 fold-changes > 1. See Supplemental Table 1 in Supplementary Material available online at http://dx.doi.org/10.1155/2015/731938 for complete test results.

### 2.11. Comparison of RNA-seq with qRT-PCR and Protein Expression

Correlation analysis was performed using all 15 samples (3 replicates for each of the 5 PDs) for MRC-5 and HFF, respectively. Spearman correlation coefficients were estimated using the RPKM values (RNA-seq data) and 2^−ΔCT^ values (qRT-PCR data). For comparison of RNA-seq with Western Blot data, only the first and the last PD were used. Log 2 fold-changes were calculated based on RPKM values (RNA-seq data), 2^−ΔΔCT^ ratios (rRT-PCR), and protein expression ratios (Western Blots).

### 2.12. Clustering of Expression Profiles

Genes were clustered according to their temporal profiles using a fuzzy *c*-means algorithm. We used the function* cmeans* from the package* e1071* 1.6-2 of the R programming language. Parameters were defined as *m* = 1.2, iter.max = 500, d.obj.fun = 10^−8^. The number of trials for the fuzzy algorithm was set to 30. The optimum number of clusters was determined using a combination of several cluster validation indexes as described by [32]. See Supplemental Table 2 for detailed assignment of the genes to clusters.

### 2.13. Functional Enrichment Analysis

Singular gene set enrichment analysis was performed using FungiFun2 [33] for selected sets of genes based on the clustering results. Although FungiFun2 is mainly suited for fungal gene enrichment analysis annotation for human genes is included as well and was recently updated. Default parameters were used while significant Gene Ontology (GO) terms and KEGG pathways were selected according to FDR corrected *p* values < 0.05. Complete lists of GO-terms and KEGG pathways are available from Supplemental Table 3. The list of GO-terms was further summarized using TreeMaps of the REVIGO online tool [34]. Default parameters and GO term with adjusted *p* values were used as input.

### 2.14. Monotonically Expressed Genes

In order to identify genes that change their expression levels monotonically with age, we calculated the Spearman correlation coefficient *c*(*i*) of each gene's temporal profile with the linearly increasing curve *f*(*x*) = *x*. In order to incorporate the replicates at each time point, we repeated the calculations by randomly sampling over the replicates at each time point and by calculating an average correlation coefficient from the resampled curves afterwards. *p* values *p*(*i*) were computed using the R base function* cor.test*. We used the calculated correlation coefficient *c*(*i*) of gene *i* with the linear increasing curve as a criterion to split the genes into the following three groups: if *c*(*i*) > 0 and *p*(*i*) < 0.05, we considered a gene to be monotonically increasing with age, if *c*(*i*) < 0 and *p*(*i*) < 0.05, the gene was considered to be monotonically decreasing with age, and if *p*(*i*) > 0.05, the expression of the corresponding gene was considered nonuniformly [35]. See Supplemental Table 4 for detailed test results.

### 2.15. Functional Association Networks

Gene symbols were used as input for functional association network creation using the STRING database [36], Cognoscente [37], and GeneMANIA [38] online tools. For STRING we used “multiple names” input and selected “Homo sapiens” as organism. In Cognoscente we selected “Human” and “Radius = 0 with intermediates” as input parameters in addition to the list of genes. The GeneMANIA network was created using default settings. The resulting networks are shown in Figure 9 and Supplemental Figure 6.

## 3. Results and Discussion

We studied the growth of two primary human fibroblast cell lines, MRC-5 and HFF, throughout their span in culture from an early PD until they achieved senescence at late PDs. Analysis of their growth behaviour (Supplemental Figure 1A) and their entry into senescence (Supplemental Figure 1B), measured by the induction of SA *β*-Gal, revealed a cell line specific transition into senescence of these two fibroblasts (MRC-5 derived from embryonic lung and HFFs derived from human foreskin). Fibroblast cell line specific growth has been observed by us before [13, 39]. Total RNA was extracted at five different time points of the fibroblasts span in culture and was subjected to high-throughput RNA sequencing (RNA-seq).

### 3.1. Global Expression Profiles Cluster according to Cell Line and Age

Overall, the RNA-seq data of this study comprise 30 samples: 15 samples for each cell line (HFF and MRC-5), consisting of five different PDs, each with three biological replicates. For each sample, mapping and counting resulted in 56,299 raw gene count expression values (using Ensembl gene annotation). The largest group of these genes (21,226) belongs to the group of protein coding genes. For all the 30 samples, 19,237 genes have raw gene counts larger than zero; these genes were considered for further analysis.

First, we studied primary clustering of the global gene expression. We therefore created a heatmap showing the Spearman correlation for all 30 samples using the nonzero genes (Figure 1). In this heatmap, both cell lines were clearly separated. In eight out of ten cases, the three values of the replicates were clustered together, showing the good quality of the data and low noise between the replicates. Next, we applied principal component analysis (PCA) to further investigate the effect of aging in the individual cell lines. Figure 2 shows the first three principal components which explain ~97% of the variances in a three-dimensional plot. MRC-5 and HFF again were clearly separated (by PC2) and the effect of aging was covered by PC1 and PC3, with a larger separation between young and old HFF compared to MRC-5. Already at this global level, similarity between both cell lines is perceptible, since young and senescent samples are grouped concordantly.

### 3.2. MRC-5 and HFF Share Common Differentially Expressed Genes Regulated by Aging

**Figure 3:**
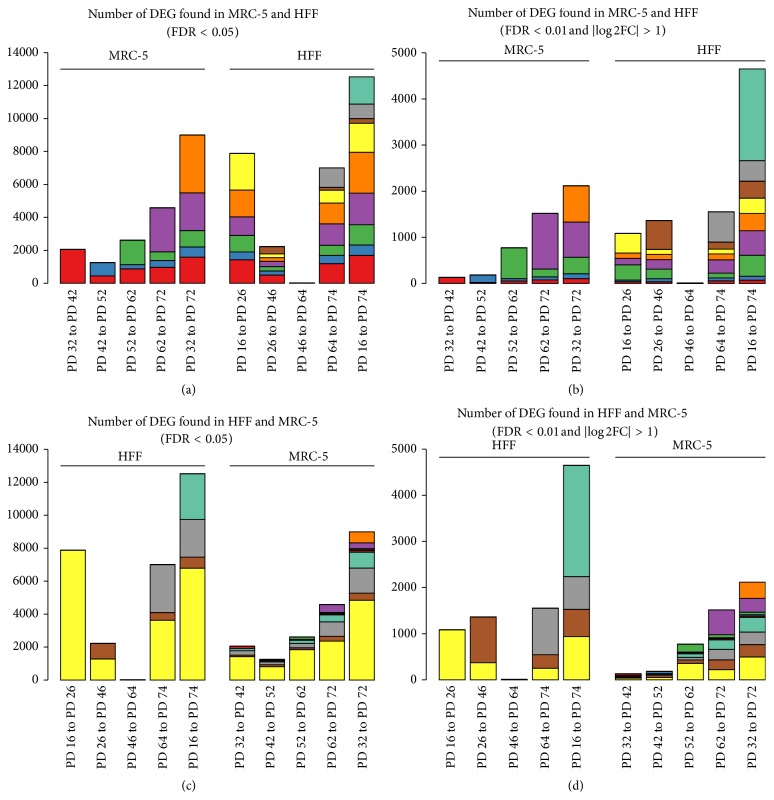
Intersection barplot showing the number of DEG between several consecutive PDs in MRC-5 and HFF for two different cutoffs ((a) + (c): FDR < 0.05; (b) + (d): FDR < 0.01 and |log 2-fold-change| > 1). In each plot, identical colors across different bars indicate the same set of DEG (intersection) while new colors indicate a new set of DEG (from left to right). (a) and (b) indicate genes which were found in MRC-5 and likewise in HFF. (c) and (d) show the same number of DEG but HFF is listed first in order to show the number of genes which were found likewise in MRC-5. For instance in (a), the red colored parts of all bars encode DEG found in MRC-5 between PD32 and PD42 while yellow colored parts of bars denote “new” DEG found in HFF between PD16 and PD26.

Differentially expressed genes (DEG) were identified by comparing all consecutive PDs as well as the first with the last PD in MRC-5 and HFF cells (10 comparisons; Table 1). Figures 3(a) and 3(c) show the absolute number of DEG found as well as the intersection of sets of DEG (indicated by color). Overall, considering all five comparisons in each cell line, we identified more DEG in HFF (14,511) compared to MRC-5 (10,517). Due to the strong effect of aging on gene expression and the large number of detected DEG, more stringent selection cutoffs (*p* < 0.01 and |log 2 fold-change| > 1) were used beyond the standard *p* value threshold of 0.05 (Figures 3(b) and 3(d)).  Figure 3 reveals that DEG were not specific for a certain PD comparison but recurred when later PDs were compared. MRC-5 and HFF shared a large fraction of DEG; only a minor fraction of DEG was identified uniquely in one of the cell lines (bars on the right in Figure 3). This indicates common processes which occur during aging in both cell lines rather than cell line specific changes. Most DEG were found when comparing the first with the last PD, leading to new DEG which had not previously been detected between consecutive PDs (orange and turquoise coloured bars in Figure 3). Both cell lines differed between the absolute number of DEG as well as the increased percentage of DEG for the first two transitions in HFF (PD 16 to PD 26; PD 26 to PD 46). In MRC-5, most of the changes seemed to occur at late PDs, while HFF cells indicated larger changes already after early PDs. This effect is also perceivable by the distances in the PCA plot (Figure 2).

### 3.3. High Correlation of RNA-seq with qRT-PCR for Selected DEG

**Figure 4:**
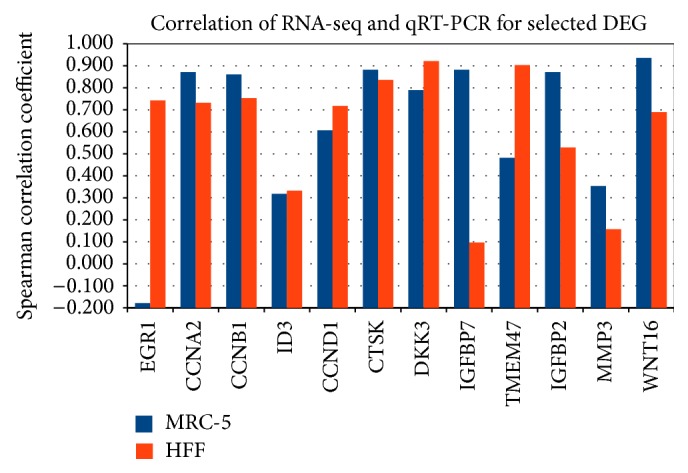
Spearman correlation of RNA-seq and qRT-PCR expression profiles for selected DEG commonly regulated in MRC-5 and HFF. Almost all genes exhibit a positive correlation between both measurement techniques (exception: EGR1 in MRC-5). With age, the first four genes (EGR1-ID3) are downregulated (first PD compared to last PD), while the later are upregulated (CCND1-WNT16).

For validation of the RNA-seq data, qRT-PCR was applied. Here, triplicates for all five PDs were measured. Selection of genes was based on the comparison of the first with the last PD, using the strict DEG criteria (*p* < 0.01 and |log 2 fold-change| > 1), resulting in 2,117 DEG for MRC-5 and 4,651 DEG for HFF (5th and 10th bars in Figures 3(b) and 3(d)). We further filtered the intersection of those two gene sets (1,139) according to common differences in both cell lines. The majority (917) of these DEG were commonly regulated, either up (385) or down (532), showing again the similarity of gene expression changes in both cell lines. Overall 12 DEG were selected which either showed strong expression in the RNA-seq data (RPKM > 50; genes:* EGR1*,* CCND1*,* CTSK*,* DKK3*,* IGFBP7*, and* TMEM47*) or were proven to have an established role in cell cycle and senescence pathways (*CCNA2*,* CCNB1*,* ID3*,* IGFBP2*,* MMP3*, and* WNT16*). The minimal RPKM criterion was applied to ensure a strong expression signal in at least one condition for a set of genes. The expression profiles from both measurement techniques were then confronted using Spearman rank correlation in each individual cell line. The results showed high correlation coefficients indicating a good overlap of both measurement techniques and quality of high-throughput gene expression analysis (Figure 4). In 17 out of 24 cases, correlation was larger than 50% (mean correlation of 63%), while only once negative correlation was found (EGR1 in MRC-5).

### 3.4. Consistent Changes of mRNA and Protein Expression

**Figure 5:**
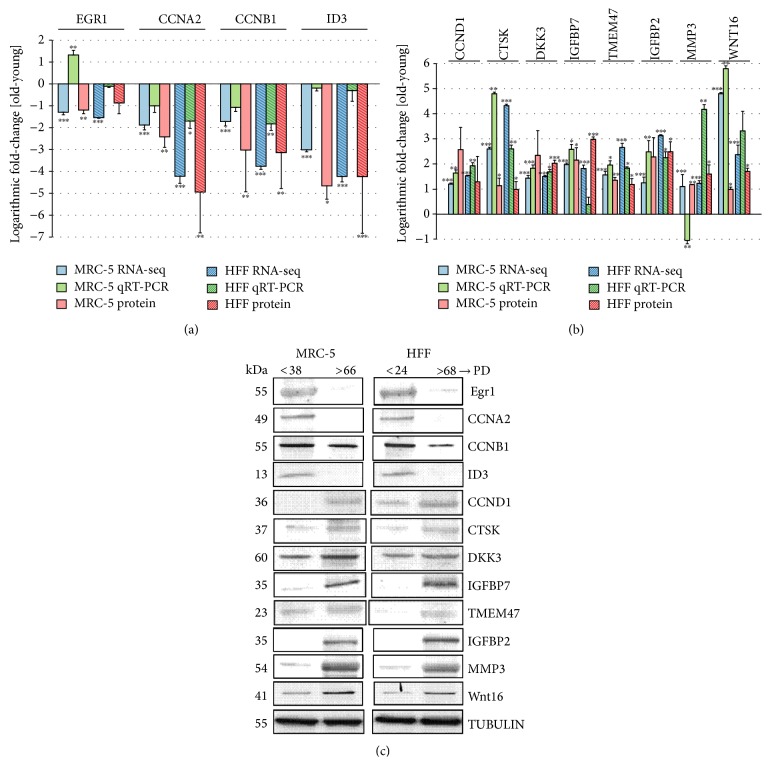
Comparison of expression changes between young and old MRC-5 and HFF fibroblasts measured by RNA-seq, qRT-PCR, and Western Blots. (a) Four genes commonly downregulated and (b) 8 genes commonly upregulated in both cell lines. (a, b) The colors of the bars indicate the measurement technique (blue: RNA-seq; green: qRT-PCR; red: Western Blots/protein expression). Solid colored bars represent MRC-5 while shaded boxes represent HFF cells. The height of the bars corresponds to the logarithmic fold-change (FC) of expression between the first and the last PD investigated here (RNA-seq: log 2 RPKM FC; qRT-PCR: log 2^−ΔΔCT^; protein: log 2 expression ratio). Error bars indicate standard deviation from the mean. Changes statistically different comparing young and old PD (RNA-seq: DESeq; rRT-PCR/Protein: Student's *t*-test; *n* = 3) are indicated with an asterisk: ^*∗*^ *p* < 0.05, ^*∗∗*^ *p* < 0.01, and ^*∗∗∗*^ *p* < 0.001. (c) The blots show the protein expression levels in MRC-5 and HFF cells at young compared to old PDs. The up- or downregulation was signified by the presence or absence of bands in Western Blots.

**Figure 6:**
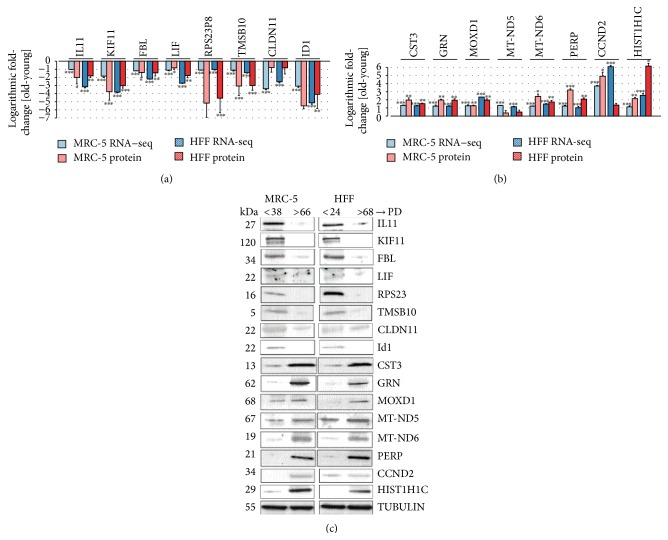
Comparison of expression changes between young and old MRC-5 and HFF fibroblasts measured with RNA-seq and Western Blots. (a) 8 genes commonly downregulated and (b) 8 genes commonly upregulated in both cell lines. (a, b) The colors of the bars indicate the measurement technique (blue: RNA-seq; red: Western Blots/protein expression). Solid colored bars represent MRC-5 while shaded boxes represent HFF cells. The height of the bars corresponds to the logarithmic fold-change (FC) of expression between the first and the last PD investigated here (RNA-seq: log 2 RPKM FC; protein: log 2 expression ratio). Error bars indicate standard deviation from the mean. Changes statistically different comparing young and old PD (RNA-seq: DESeq; rRT-PCR/Protein: Student's *t*-test; *n* = 3) are indicated with an asterix: ^*∗*^ *p* < 0.05, ^*∗∗*^ *p* < 0.01, and ^*∗∗∗*^ *p* < 0.001. (c) The blots show the protein expression levels in MRC-5 and HFF cells at young compared to old PDs. The up- or downregulation was signified by the presence or absence of bands in Western Blots.

Although mRNA expression changes are generally considered to consequently lead to corresponding changes in protein levels, correlation between both can be as little as 40%, as observed in large-scale proteome- and transcriptome-profiling experiments [40]. We thus asked if the detected changes of strongly altered DEG correlated with corresponding protein expression levels. Triplicates of the first and last PD were selected for comparison. Gene selection was performed as described above, either by strong expression in RNA-seq (RPKM > 35) or by functional relation to cell cycle and senescence pathways. Overall, 28 DEG (16 down- and 12 upregulated DEG) were selected (the genes mentioned above, validated by qRT-PCR, were included in this set). The results of this comparison showed consistent changes, in terms of their direction of regulation, between mRNA expression, measured by RNA-seq, and protein expression, measured by Western Blots, for all selected genes (Figures 5 and 6). 44 out of the 56 protein fold-changes exhibited significant differences between young and old PDs.

### 3.5. Common Genes Ruling the Transition into Senescence in MRC-5 and HFF

Then, we asked if common cellular markers are involved in the transition into senescence. We thus studied the genes most differentially expressed with age commonly in MRC-5 and HFF fibroblasts. We noticed that a large number of genes among the most differentially expressed genes belonged to the secretory phenotype (Figure 8(a), as explained in Section 3.7). The list of genes included* CTSK*, normally stimulated by inflammatory cytokines released after tissue injury [41],* GRN*, a previously functionally validated gene responsible for wound healing [42],* CST3*, associated with sarcopenia [43], and* PERP*, a p53 apoptosis effector, the mRNA expression level of which is upregulated in human mesenchymal stem cells [44]. We detected significant upregulation of* IGFBP2* which was found upregulated with senescence in retinal pigment epithelial cells [45, 46] and BJ fibroblasts [47].* ID1*,* ID3*,* CCNA2,* and* CCNB1* showed significant downregulation with age in our study for both human fibroblasts. Downregulation of* ID1* and* ID3* expression with senescence was detected in BJ foreskin, WS1 fetal skin, and LF1 lung human fibroblasts [48] and of* CCNA2* in IMR-90 and WI-38 [49]. Targeting* CCNB1* expression inhibits proliferation of breast cancer cells [50]. The list of most differentially expressed genes also included* IGFBP7* and* MMP3* which encode protein receptors predominantly located on the cell surface. Both* IGFBP7* and* MMP3* are upregulated with senescence in human melanocytes [51–54]. Recently we found that overexpression of recombinant IGFBP7 proteins induced premature senescence in early PD MRC-5 fibroblasts [13].

Among the genes significantly upregulated with age in both MRC-5 and HFFs we identified* DKK3*, having a role in Wnt signaling [55–57]. DKK3 has tumor suppressor activity in breast cancer patients [58] and in papillary thyroid carcinoma [57]. However, we had failed to demonstrate an induction of premature senescence in early PD HFFs on overexpression of recombinant DKK3 proteins [13]. Though not significantly differentially expressed with age in MRC-5 fibroblasts, one of the genes which were most significantly upregulated with age in HFFs was the* SFRP4* gene, an antagonist for Wnt signalling [59]. SFRP4 acts as a tumor suppressor in gastric carcinoma [60] and epithelial ovarian cancer cell lines [61]. In a separate study, we functionally validated the expression of* SFRP4* in early PD HFF and MRC-5 fibroblasts by treating them separately with human recombinant SFRP4 protein. This treatment resulted in premature senescence induction in HFFs but not in early PD MRC-5 fibroblasts [13]. Here, induction of SFRP4 mRNA expression was not detected by RNA-seq, explaining the lack of premature senescence induction in early PD MRC-5 fibroblasts. SFRP4 expression thus showed cell line specific differences.

### 3.6. Clustering of the Expression Profiles Shows Similar Pattern in Both Cell Lines

**Figure 7:**
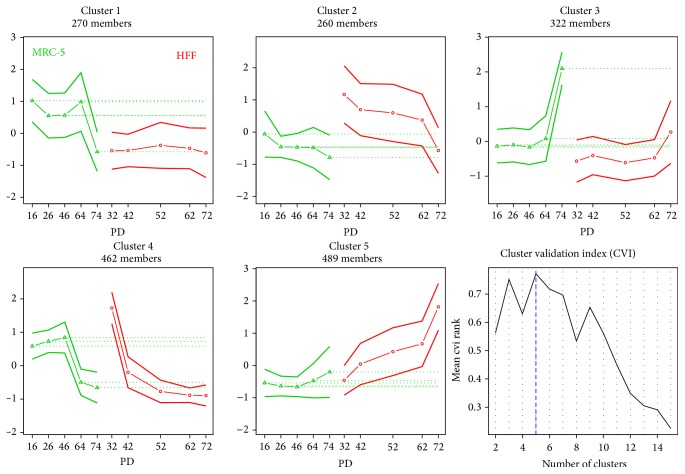
Combined line plot showing the scaled and clustered expression profiles of 1,803 genes detected in both cell lines as differentially expressed. The middle line, including the points, indicates the mean expression profile for all included genes in a certain cluster. The thicker lines above and below indicate the standard deviation. Cell lines are indicated by color (green lines: MRC-5; red lines: HFF cells). The horizontal dotted green lines can be used to compare the mean profiles of both cell lines. The last plot shows a summary of the cluster validation analysis. An optimal number of five clusters were estimated.

We found many differentially expressed genes commonly regulated in both MRC-5 and HFF. Next, we asked if both cell lines exhibit common temporal expression profiles rather than showing different effects for the same set of genes. Therefore, we applied fuzzy *c*-means clustering comparing the expression profiles of both cell lines. We used 1,803 genes found to be differentially regulated between the four consecutive PDs and between the first and the last PD in both cell lines, according to the strict cutoffs as shown in Figures 3(b) and 3(d) (FDR < 0.01; |log 2 fold-change| > 1). Using several cluster validation indexes, an optimal number of five clusters were estimated, and each selected DEG was assigned to one out of these five groups (Figure 7). The majority of DEG exhibits similar temporal expression profiles in MRC-5 and HFF. 811 DEG were upregulated (clusters 3 and 5) and 722 are downregulated (clusters 2 and 4). Stronger differences between both cell lines were found for genes grouped in clusters 1 and 4. Interestingly, most genes follow a monotonic profile (either up or down) while only few genes exhibit a parabolic-like shape. Clusters 1 and 3 show major changes in MRC-5 between the second to last and the last PD, while cluster 4 groups genes with large differences between the first and the second PD in HFF. This effect was already observed when comparing DEG between the consecutive PDs (see DEG section above). Figure 7 summarizes the gene expression profiles by showing only the scaled and centred mean and standard deviation of the DEG clustered. In most of the cases, the absolute expression values were different between both cell lines (indicated by the dashed horizontal lines) but the trends of the actual changes across the five PDs were similar. For instance, cluster 3 contains genes which show larger mean expression values for MRC-5 but are upregulated with increasing PDs in both cell lines (*vice versa* in cluster 2).

### 3.7. Identification of Functional Categories Significantly Enriched for Genes with Common Expression Profiles

**Figure 8:**
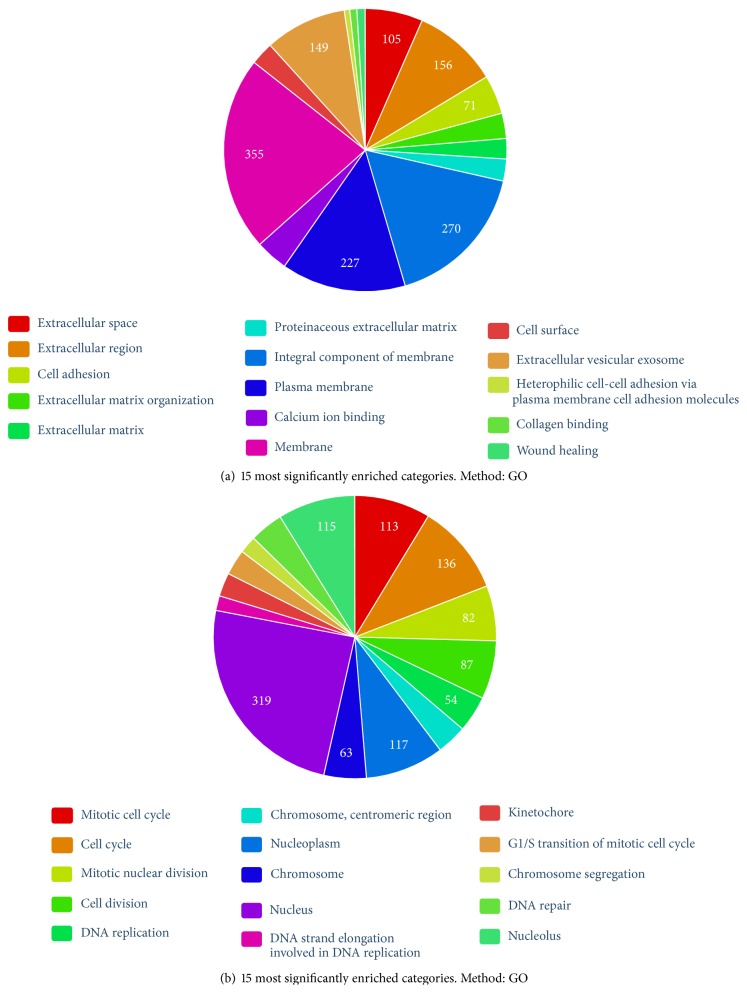
(a) Top 15 of 171 significantly enriched GO-terms based on 811 upregulated genes (clusters 3 and 5). (b) Top 15 of 253 significantly enriched GO-terms based on 722 downregulated genes (clusters 2 and 4). The numbers in the pie-chart correspond to the number of genes which were included in the clusters and assigned to the GO-terms stated in the chart.

Next, we deduced the main biological processes driven by the differentially expressed gene sets obtained from the cluster analysis. Using gene set enrichment analysis, for each of the five clusters significant GO categories and KEGG pathways could be identified. The results indicated a strong connection of upregulated genes (grouped in clusters 3 and 5) to “extracellular space” (GO:0005615) and “membrane” (GO:0016020) components (Figure 8(a)). Corresponding KEGG pathways, found for these genes, were for example, “ECM-receptor interaction” (hsa04512) and “ABC transporters” (hsa02010; see Supplemental Table 3). ABC proteins transport various molecules across extra- and intracellular membranes and are involved in aging and age-related diseases [62]. Cluster 3 shows stronger upregulation at late PD for MRC-5 cells while HFF cells are upregulated more clearly in cluster 5 (see above). Comparing the GO-terms found for these single clusters, we found links of the stronger upregulation in MRC-5 with “integral component of plasma membrane” (GO:0005887) and the “Golgi apparatus” (GO:0005794), while “sarcolemma” (GO:0042383) and “nucleosome” (GO:0000786) were more specific for upregulation in HFF (Supplemental Figure 2). The structure of the secretion regulating Golgi complex is altered in senescent cells [63]. While our results indicate cell line specific differences during replicative senescence, the GO-term comparison revealed that in both cell lines many genes were similarly upregulated. A large set of GO-terms associated with upregulated genes were related to the senescence associated secretory phenotype [64].

Downregulated genes (grouped in clusters 2 and 4) were associated to strongly enriched GO processes related to, for example, “cell cycle” (GO:0007049), “cell division” (GO:0051301) and “DNA replication” (GO:0006260) (Figure 8(b)). Here, differences between both cell lines are more obvious. After a slight initial gain, expression in MRC-5 cells declined strongly between PD 46 and PD 64. In HFFs, strong decline already started after PD32 without larger changes for late PD (Figure 7; cluster 4). Most of these cell cycle related genes, which account for the above-mentioned profiles, are related to the cellular component “nucleoplasm” (GO:0005654). Associated GO-terms for cluster 2, which depicts moderate downregulation, were more widespread and covered processes like “positive regulation of nitric oxide biosynthetic process” (GO:0045429), “endoderm formation” (GO:0001706), and “response to cAMP” (GO:0051591; Supplemental Figure 3).

Cluster 1 showed the largest differences between both cell lines. While, in MRC-5, genes are downregulated strongly at the last PD, no clear up- or downregulation is observed for HFF. Significantly enriched GO-terms associated to these genes were, for example, “vasculogenesis” (GO:0001570), “response to lipopolysaccharide” (GO:0032496), and “cell adhesion” (GO:0007155) (Supplemental Figure 4).

### 3.8. Monotonically Regulated Genes in MRC-5 and HFF Are Connected in Functional Association Networks

Since senescence is a continuous cellular process, it can be hypothesized that genes possessing key relevance for senescence change their expression values monotonically over time, while genes with irregular temporal expression patterns might be associated with response to environmental conditions, with the circadian rhythm or other processes.

Amongst others, continuous increasing and decreasing profiles were found by the clustering analysis. In addition to this nonbiased approach, we intended to identify genes with a strong monotonic behaviour across the PDs investigated here. We calculated the Spearman correlation coefficient of each gene's temporal profile with a linearly increasing sequence. Replicates for each PD were incorporated by a random sampling approach. Subsequently, we classified genes into three classes according to their behaviour with age: (a) monotonically upregulated genes, (b) monotonically downregulated genes, and (c) nonuniformly regulated genes (Table 2).

**Figure 9:**
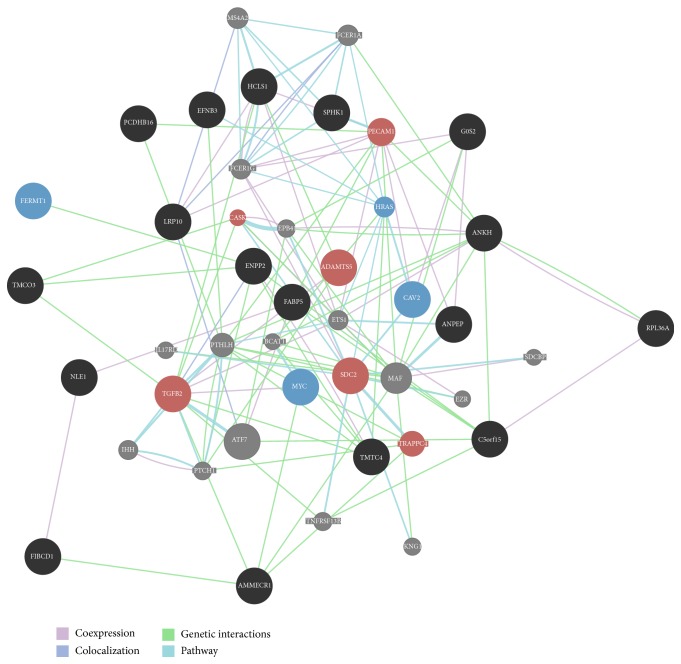
Functional association network by GeneMANIA for 23 monotonically up- or downregulated genes. Input genes are depicted by either black, red, or blue color or large circles. Intermediate genes, added by the tool, are shown using small grey circles. Colored genes denote functions associated by the tools: blue = “epithelial cell proliferation”; red = “ extracellular matrix organization.” The edge colors indicate the type of interaction, as explained in the legend on the bottom right.

More monotonically up- and downregulated genes were found for HFFs compared to MRC-5 (888 versus 179). Only a small subset of these genes were commonly regulated in both cell lines (9 up and 14 down) but even less genes showed an opposite monotonic expression profiles (8; see Supplemental Figure 5 and Supplemental Table 4). The 23 commonly monotonically up- or downregulated genes were studied in more detail. Since in both cell lines the regulation of these genes strongly correlated with an increase of senescence, they might play an essential role in cellular aging and may rule common regulatory process. We used several online resources in order to find potential or validated interactions between these genes. The STRING database [36] only provides the interactions between four out of all the 23 genes (Supplemental Figure 6A). Using Cognoscente [37], 17 out of 23 genes were connected within one interaction graph (Supplemental Figure 6B). More interactions could be found using GeneMANIA [38], leading to a network which is widely connected by coexpression and common pathways like, for example, “epithelial cell proliferation” and “extracellular matrix organization” (Figure 9). Both of the latter tools integrate intermediate genes which were not in the input list. Hub genes in these networks included* ATF7*,* MAF*,* UBC,* and* ELAVL* which are interesting candidates for further studies. All the four of these genes were functionally associated with tumorigenesis. Members of the ubiquitin family including UBC have been associated with tumor progression [65]. In terms of ATF7, the activating transcription factor family is associated with cell proliferation and oncogenesis [66]. Both MAF and ELAV1 have been associated with oncogenesis and tumor progression [67, 68]. Thus all the four genes had an association with cell proliferation. Then, we investigated the biological relevance of the monotonically up- and downregulated genes in both fibroblast cell lines. The list of monotonically downregulated genes included* NLE1*,* AMMECR1*,* FIBCD1*,* ENPP2*,* TMTC4*,* ANPEP*,* MYC*,* EFNB3*,* HCLS1*,* FERMT1*,* FABP5*,* SPHK1*,* GOS2,* and* RPL36A*. The genes monotonically upregulated included* LRP10*,* TMCO3*,* CAV2*,* ADAMTS5*,* C5orf15*,* SDC2*,* ANKH*,* PCDHB16,* and* TGFB2*. A number of genes in the above list have been functionally associated with proliferation.

#### 3.8.1. Monotonically Downregulated Genes

*NLE* plays a role in regulating the Notch activity and is involved in embryonic development in mammals by affecting the CDKN1A and Wnt pathways [69]. Forced expression of miR-26 inhibits the growth of stimulated breast cancer cells and tumor in xenograft models by reducing the mRNA expression levels of AMMECR1 and other genes [70]. AMMECR1 is associated with Alport syndrome, mental retardation, midface hypoplasia, and elliptocytosis [71]. FIBCD1 (fibrinogen C domain containing 1) binds to chitin of invading parasites [72]. FIBCD1 is primarily present in the gastrointestinal tract of humans; however, their presence in skin has been highly debated [73, 74]. ENPP2 facilitates cell motility and progression and is related to the invasion of ductal breast carcinomas [75].* TMTC4* is a gene contributing to embryonic brain development; it interacts with Wntless, an integral Wnt regulator [76].* EFNB3*, a member of the ephrin gene family, is associated with neural development [77]. ANPEP is a well-known marker for acute myeloid leukemia and tumor invasion; it has a regulatory role in angiogenesis [78, 79]. FERMT1 is overexpressed in colon and lung carcinomas [80]. The* MYC* oncogene is associated with cell growth regulation by driving proliferation via upregulation of Cyclins and downregulation of p21 [81, 82].* HCLS1* gene which is monotonically downregulated with age is associated with antigen receptor signaling and clonal expansion as well as deletion of lymphoid cells [83]. The* FABP5* gene encodes the fatty acid binding protein in epidermal cells and is upregulated in psoriatic tissues [84]. SPHK1 has been previously associated with melanoma progression and angiogenesis [85, 86]. The* GOS2* gene promotes apoptosis by binding to BCL2, hence preventing the formation of protective BCL2-BAX; its mRNA and protein levels are downregulated in type 2 diabetic patients [87, 88]. Thus, almost all genes, monotonically downregulated with age in both fibroblast cell lines, are associated with proliferation and cell survival.

#### 3.8.2. Monotonically Upregulated Genes

*LRP10*, a negative regulator of Wnt signalling, was found monotonically upregulated with age [89]. CAV2 is a scaffolding protein within the caveolar membrane modulating cancer progression [90]. ADAMTS5 enables the destruction of aggrecan in patients with arthritic disease which is prevalent with aging [91]. The* ANKH* gene, associated with regulation of tissue calcification and in turn susceptibility to arthritis, is also monotonically upregulated with age in both fibroblast cell lines [92]. Syndecan-2 protein (SDC2) is upregulated in skin and lung tissues of patients suffering from (age-associated) systemic sclerosis and fibrosis [93, 94]. mRNA expression of PCDHB16 is upregulated in patients with (age-associated) Alzheimer's disease [95]. TGFB2, also monotonically upregulated with age in fibroblasts, has suppressive effects on interleukin-2 dependent T cell proliferation and displays effector functions [96].

In summary, the genes, which we found here monotonically up- and downregulated with age in both fibroblast cell lines, have been studied before. In this study, we explicitly show for the first time the age-associated regulation of these genes in primary human fibroblast cells of two different origins. In a following study we will determine the protein expression of all age-related genes and functionally validate the expression of these genes.

## 4. Conclusion

We studied molecular aspects of cellular aging by determining the differential expression of genes during the aging of two primary human fibroblasts, MRC-5 and HFFs. RNA-seq data analysis encompassed different levels, starting from the complete set of annotated and expressed genes, proceeding to different gene subsets and functional categories. Most of the detected changes were found to be common in both cell lines, as indicated by the large number of overlapping DEG and common expression profiles identified by clustering. We validated the expression patterns for selected genes, demonstrating an association of almost all most differentially expressed genes with proliferation or cell cycle arrest, consistent with previous senescence studies. Investigating expression changes across five consecutive PDs and comparing young with senescent cells enabled us to identify both monotonically up- and downregulated genes as well as the most differentially expressed genes. Both sets of genes strongly contributed to the transition into cellular senescence. Thus, we quantitatively describe similarities in gene expression profiles during the aging of two fibroblast cell lines of different origin.

## Data Deposition

The RNA-seq data discussed in this publication have been deposited in NCBIs Gene Expression Omnibus and are accessible through GEO Series accession number GSE63577.

## Supplementary Material

Suppl. Figure 1: (A) Growth curve of MRC-5 and HFF fibroblasts derived from a single vial and maintained in culture as triplicates from an early PD until senescence at late PDs. Data points of all measurements are displayed (not the mean). PDs specified in the plot denoted the time points were the total RNA was collected and subjected to RNA-seq. Black line denotes MRC-5 and green denotes HFF (B) Percentage of SA-β Gal positive cells at different time points of their growth in culture in MRC-5 and HFF fibroblasts. Each curve is measured in triplicate, the mean value is displayed with error bar (± S.E). Red line denotes MRC-5 and black denotes HFF.
Suppl. Figure 2: TreeMaps produced using REVIGO () summarizing: (A) 12 GO cellular component (CC) terms found for cluster 3. (B) 21 GO CC terms found for cluster 5. Both clusters contain up-regulated genes but exhibit differences between MRC-5 and HFF.
Suppl. Figure 3: TreeMaps produced using REVIGO () summarizing: (A) 59 GO biological processes (BP) terms found for cluster 2. (B) 162 GO BP terms found for cluster 4. Both clusters contain down-regulated genes.
Suppl. Figure 4: TreeMaps produced using REVIGO () summarizing 24 GO BP terms found for cluster 1.
Suppl. Figure 5: Venn plots showing the number of detected significantly monotonically expression pattern. (A, B): up-/down-regulated in both MRC-5 and HFF. (C) up-regulated in MRC-5 and down-regulated in HFF. (D): vice versa of (C).
Suppl. Figure 6: Functional association networks generated using (A) STRING DB and (B) Cognoscente including 23 genes found to be monotonically expressed across the five PD. (A) Only connections between four genes were found using STRING DB. Different line colors represent the types of evidence for the association, explained on the right. (B) Nodes outlined in red are the 23 input genes. There were no interactions found for PCDHB16. 19 intermediates were included by the tool. The edge colors indicate the type of interaction, as explained in the legend on top.
Supplementary Table 1: Excel table with detailed results of tests for differential expression (DESeq, edgeR). The file contains 10 sheets: sheet 1-5: 5 comparisons for MRC-5; sheet 6-10: 5 comparisons for HFF (see Table 1). Each Sheet contains 15 columns and the list of DEG as rows. Only genes with FDR < 0.05 by DESeq and edgeR are listed.
Column 1-4: gene annotation.Column 5-10: RPKM values for each single sample.Column 11-13: mean RPKM values and log2 fold-change.Column 14-15: adjusted p-values (FDR) by DESeq and edgeR.
Supplementary Table 2: Excel table with fuzzy c-mean clustering results.
Column 1-4: gene annotation.Column 5-14: mean RPKM values of MRC-5 and HFF and all five PD.Column 15: cluster membership number [1-5].
Supplementary Table 3: Excel table with functional enrichment analysis (FungiFun). The file contains 9 sheets: One sheet for each single cluster used for GO enrichment analysis; two sheets with GO results for combined clusters; and two sheets with KEGG results for combined clusters. Each sheet contais the pathway/term IDs, the corresponding names, toplevels, exact p-values, adjusted p-values, number of genes per pathway/term and per input list.
Supplementary Table 4: Excel table with detailed results from the monotony test. Commonly significantly found genes are highlighted by red and green background color. 
Column 1-3: gene annotation.Column 4-5: Spearman correlation values and p-values (cor.test) for MRC-5.Column 6-7: Spearman correlation values and p-values (cor.test) for HFF.

## Figures and Tables

**Table 1:** Number of DEG across different PD in MRC-5 and HFF for two significance criteria. See Supplemental Table 1 for detailed test results.

Comparison	Number of DEG (FDR < 0.05)	Number of DEG (FDR < 0.01; |log⁡2FC| > 1)
MRC5: PD 32 to PD 42	2,050	131
MRC5: PD 42 to PD 52	1,248	185
MRC5: PD 52 to PD 62	2,617	773
MRC5: PD 62 to PD 72	4,582	1,516
MRC5: PD 32 to PD 72	8,992	2,117
HFF: PD 16 to PD 26	7,873	1,083
HFF: PD 26 to PD 46	2,228	1,366
HFF: PD 46 to PD 64	15	8
HFF: PD 64 to PD 74	7,002	1,553
HFF: PD 16 to PD 74	12,529	4,651

**Table 2:** Number of genes whose expression values are monotonically up- and downregulated, respectively, for MRC-5 and HFF cells. Note that monotonic behaviour does not necessarily include differential expression. See Supplemental Table 4 for complete test results.

Class	MRC-5	HFF
Monotonically upregulated	47	423
Monotonically downregulated	132	465
Nonuniformly (not significant)	19,058	18,349
