# Supplementary material for: Similarities in Gene Expression Profiles during In Vitro Aging of Primary Human Embryonic Lung and Foreskin Fibroblasts
Source: Biomed Res Int. 2015 Aug 3;2015:731938. doi: 10.1155/2015/731938 (PMC4538583; doi:10.1155/2015/731938)
Supplement: Supplementary file 1 — Suppl. Figure 1: (A) Growth curve of MRC-5 and HFF fibroblasts derived from a single vial and maintained in culture as triplicates from an early PD until senescence at late PDs. Data points of all measurements are displayed (not the mean). PDs specified in the plot denoted the time points were the total RNA was collected and subjected to RNA-seq. Black line denotes MRC-5 and green denotes HFF (B) Percentage of SA-β Gal positive cells at different time points of their growth in culture in MRC-5 and HFF fibroblasts. Each curve is measured in triplicate, the mean value is displayed with error bar (± S.E). Red line denotes MRC-5 and black denotes HFF. Suppl. Figure 2: TreeMaps produced using REVIGO () summarizing: (A) 12 GO cellular component (CC) terms found for cluster 3. (B) 21 GO CC terms found for cluster 5. Both clusters contain up-regulated genes but exhibit differences between MRC-5 and HFF. Suppl. Figure 3: TreeMaps produced using REVIGO () summarizing: (A) 59 GO biological processes (BP) terms found for cluster 2. (B) 162 GO BP terms found for cluster 4. Both clusters contain down-regulated genes. Suppl. Figure 4: TreeMaps produced using REVIGO () summarizing 24 GO BP terms found for cluster 1. Suppl. Figure 5: Venn plots showing the number of detected significantly monotonically expression pattern. (A, B): up-/down-regulated in both MRC-5 and HFF. (C) up-regulated in MRC-5 and down-regulated in HFF. (D): vice versa of (C). Suppl. Figure 6: Functional association networks generated using (A) STRING DB and (B) Cognoscente including 23 genes found to be monotonically expressed across the five PD. (A) Only connections between four genes were found using STRING DB. Different line colors represent the types of evidence for the association, explained on the right. (B) Nodes outlined in red are the 23 input genes. There were no interactions found for PCDHB16. 19 intermediates were included by the tool. The edge colors indicate the type of interaction, as explained in [file 731938.f1.zip › mat.731938.v2/Hindawi Suppl figures_230115.pdf]

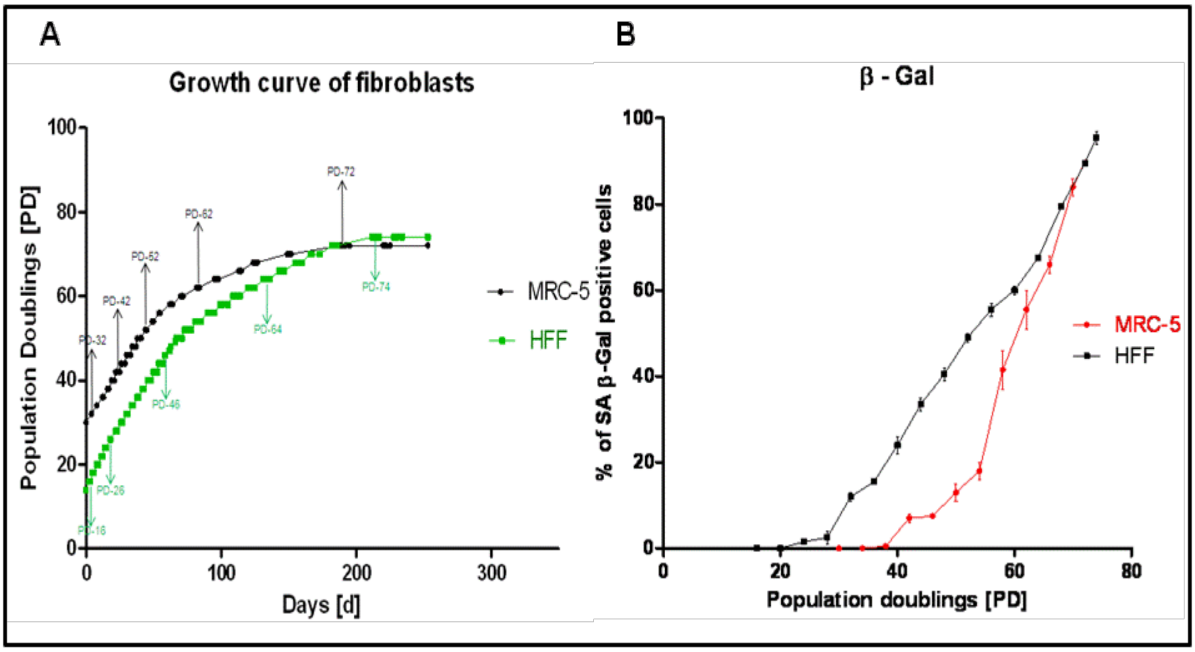

Suppl. Figure 1: (A) Growth curve of MRC-5 and HFF fibroblasts derived from a single vial and maintained in culture as triplicates from an early PD until senescence at late PDs. Data points of all measurements are displayed (not the mean). PDs specified in the plot denoted the time points were the total RNA was collected and subjected to RNA-seq. Black line denotes MRC-5 and green denotes HFF **(B)** Percentage of SA-β Gal positive cells at different time points of their growth in culture in MRC-5 and HFF fibroblasts. Each curve is measured in triplicate, the mean value is displayed with error bar (± S.E). Red line denotes MRC-5 and black denotes HFF.

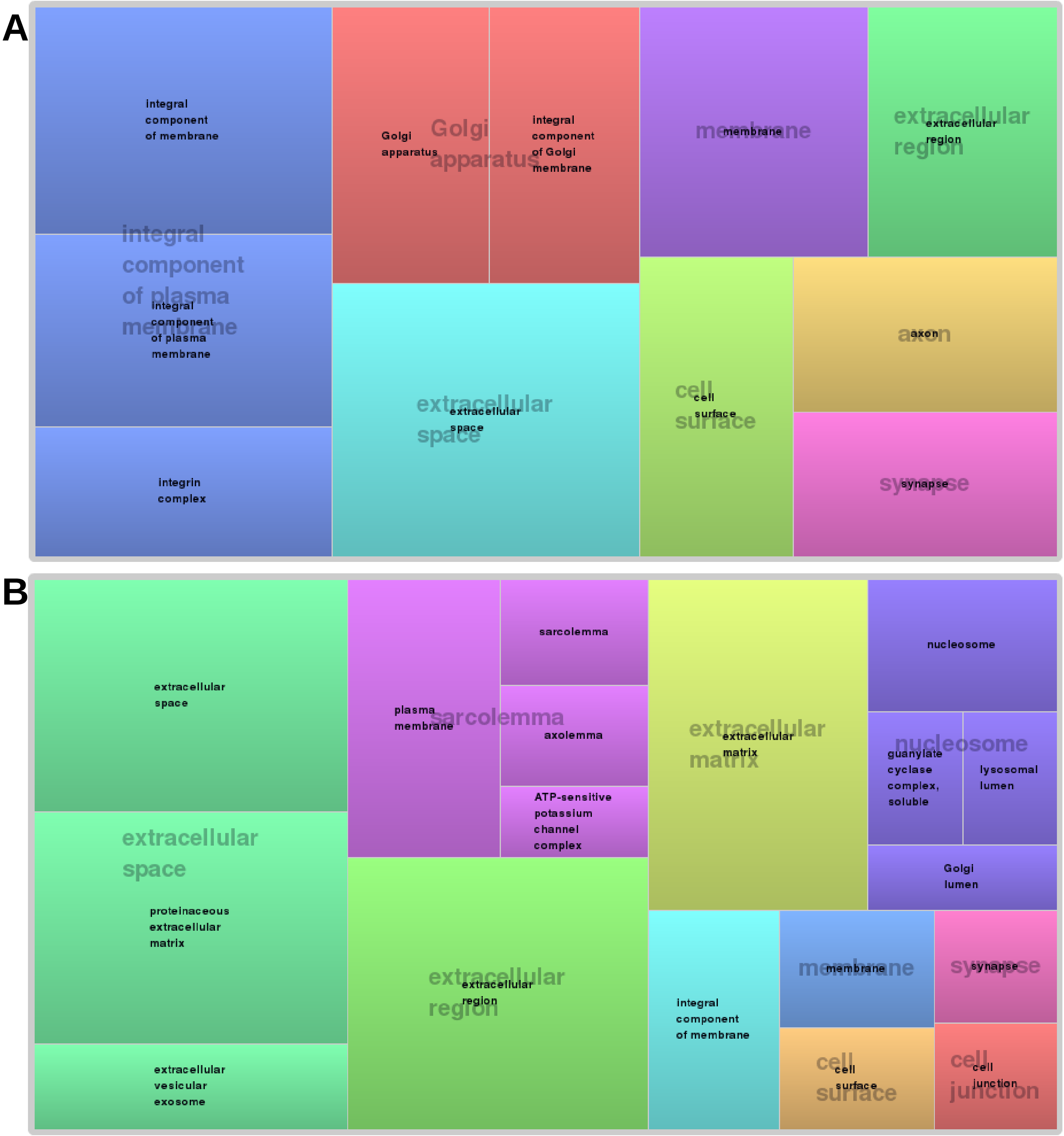

Suppl. Figure 2: TreeMaps produced using REVIGO (http://revigo.irb.hr) summarizing: (A) 12 GO cellular component (CC) terms found for cluster 3. (B) 21 GO CC terms found for cluster 5. Both clusters contain up-regulated genes but exhibit differences between MRC-5 and HFF.

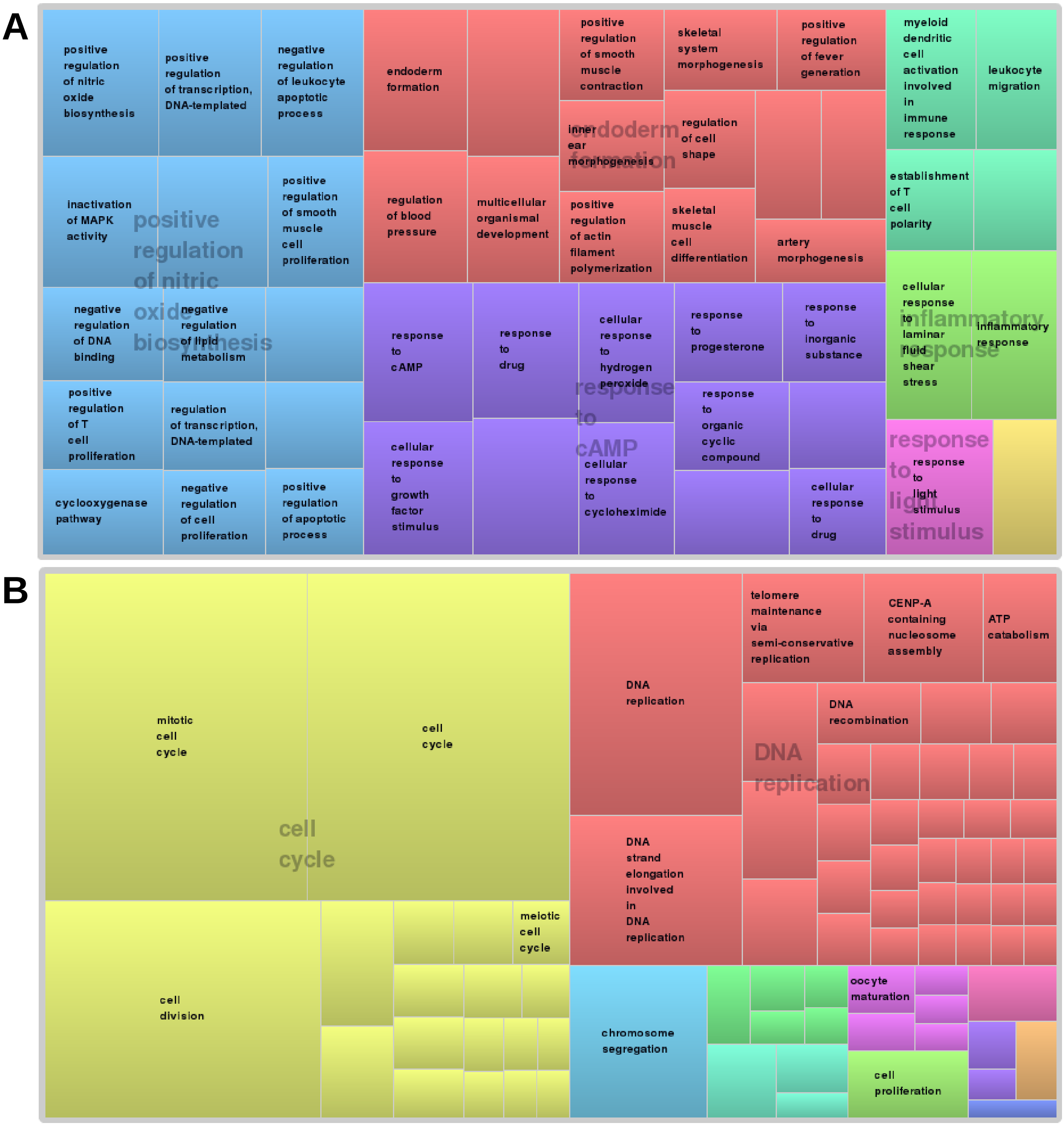

Suppl. Figure 3: TreeMaps produced using REVIGO (http://revigo.irb.hr) summarizing: (A) 59 GO biological processes (BP) terms found for cluster 2. (B) 162 GO BP terms found for cluster 4. Both clusters contain down-regulated genes.

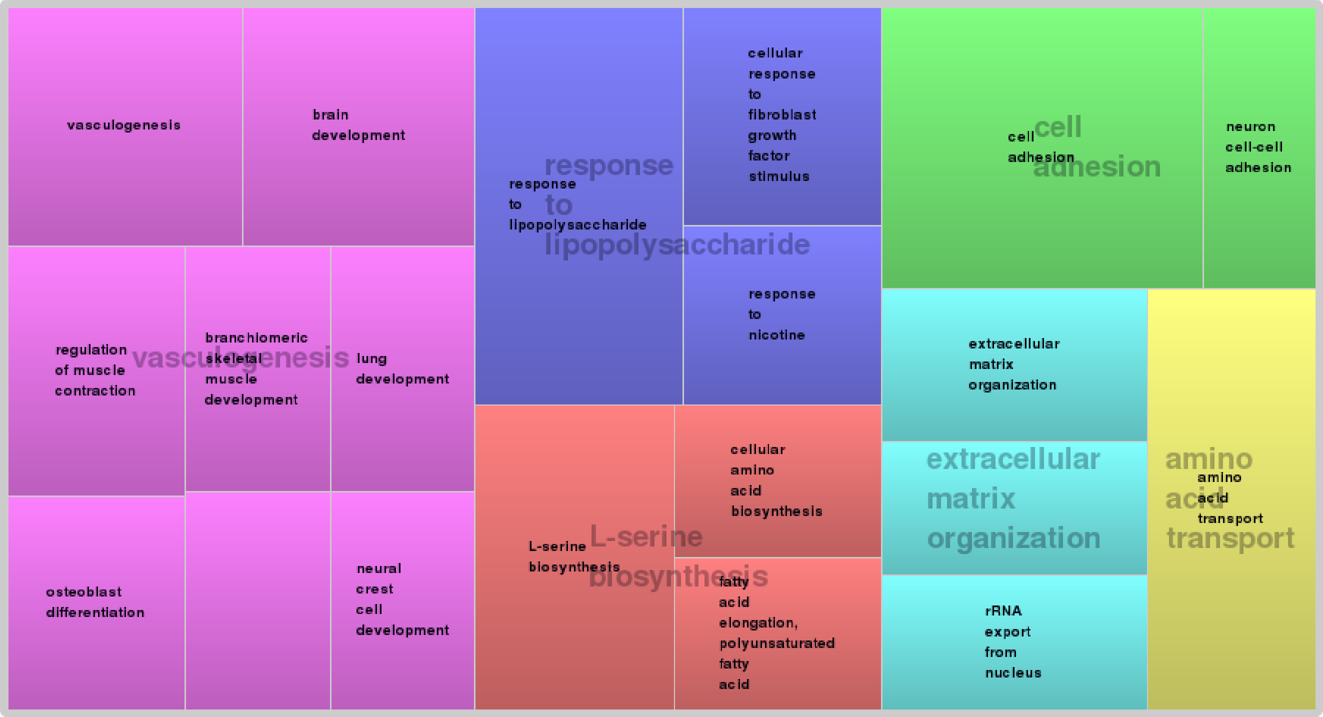

Suppl. Figure 4: TreeMaps produced using REVIGO (http://revigo.irb.hr) summarizing 24 GO BP terms found for cluster 1.

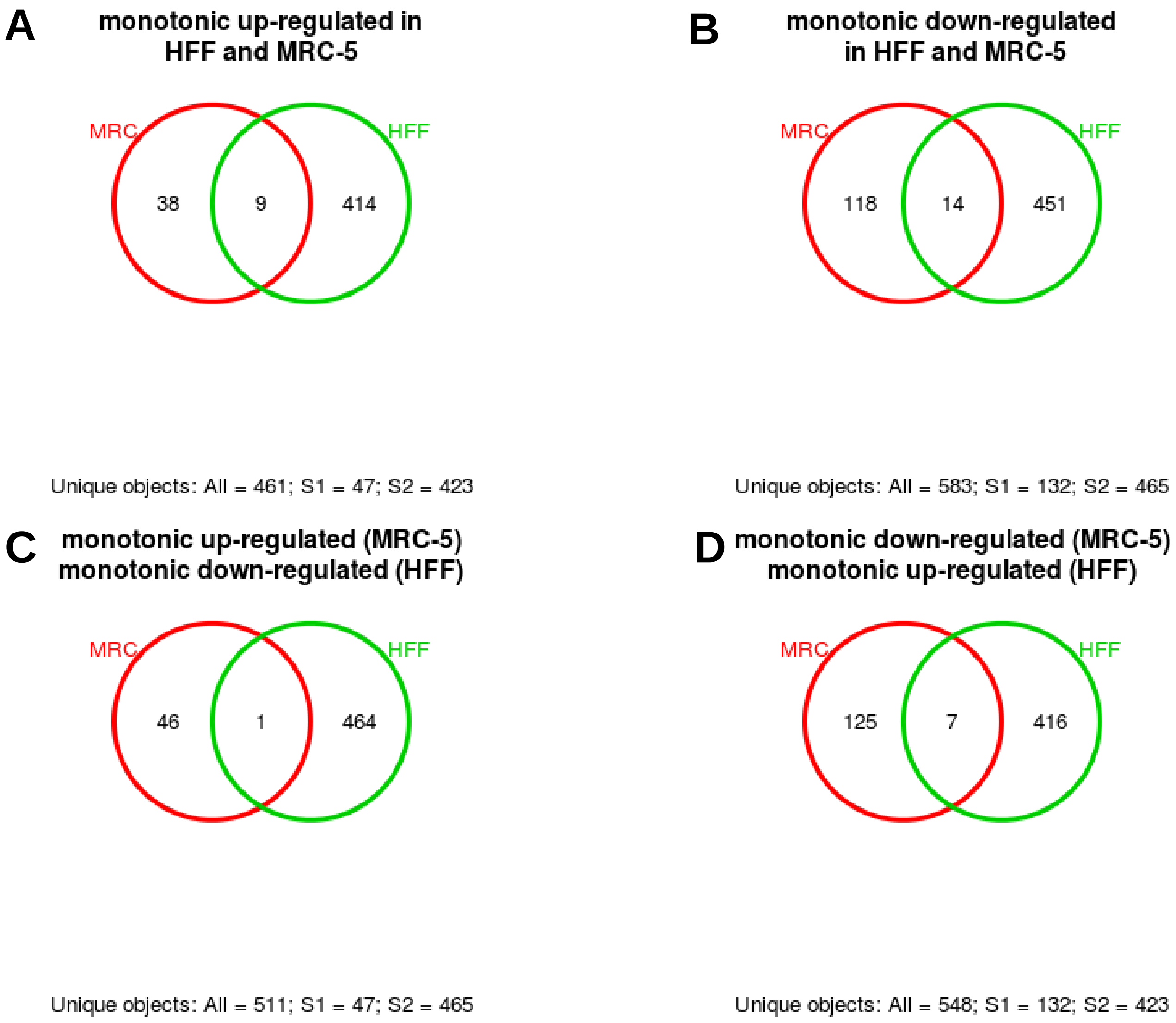

Suppl. Figure 5: Venn plots showing the number of detected significantly monotonically expression pattern. (A, B): up-/down-regulated in both MRC-5 and HFF. (C) up-regulated in MRC-5 and down-regulated in HFF. (D): *vice versa* of (C).

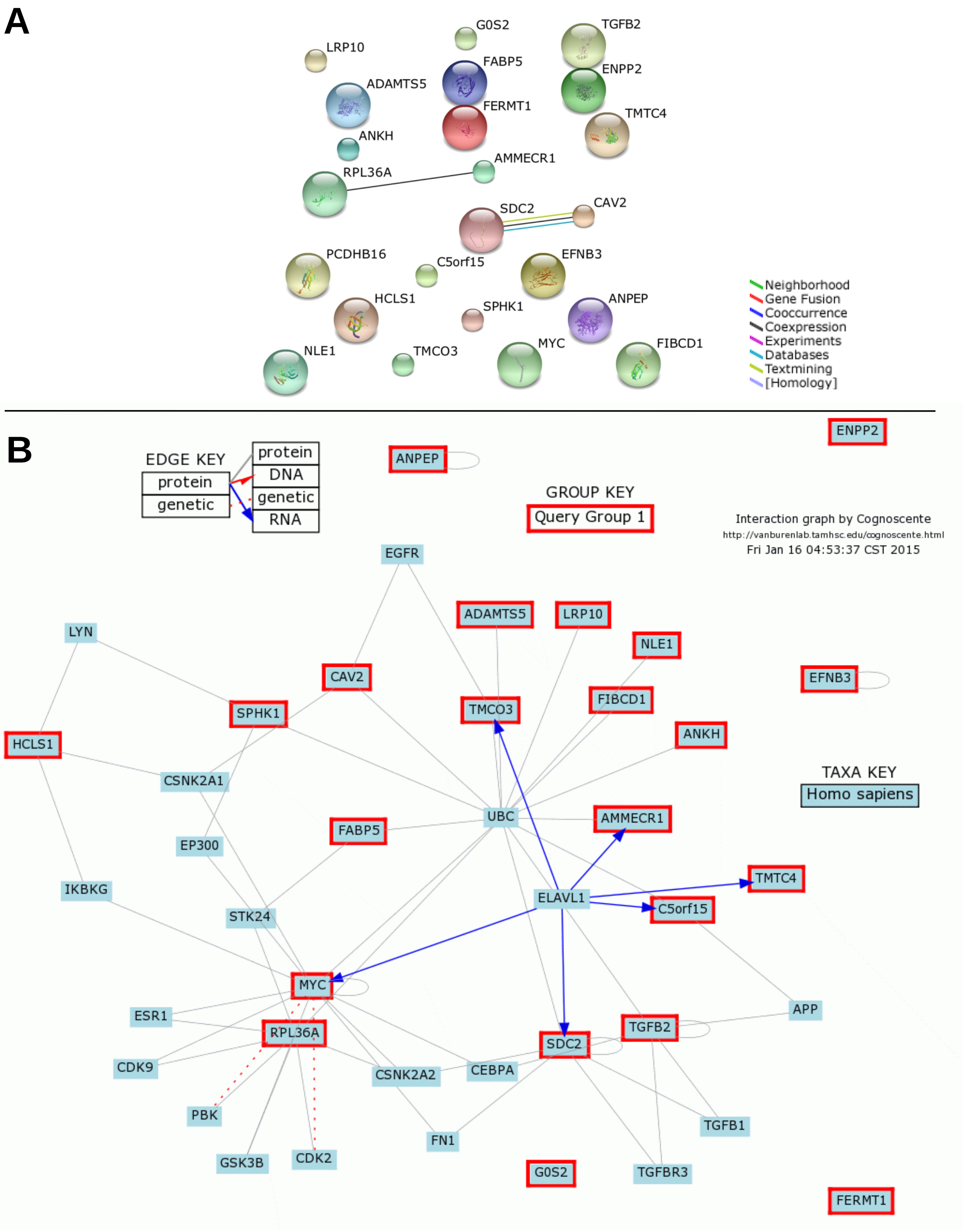

Suppl. Figure 6: Functional association networks generated using (A) STRING DB and (B) Cognoscente including 23 genes found to be monotonically expressed across the five PD. (A) Only connections between four genes were found using STRING DB. Different line colors represent the types of evidence for the association, explained on the right. (B) Nodes outlined in red are the 23 input genes. There were no interactions found for PCDHB16. 19 intermediates were included by the tool. The edge colors indicate the type of interaction, as explained in the legend on top.

Supplementary Table 1:
Excel table with detailed results of tests for differential expression (DESeq, edgeR). The file contains 10 sheets: sheet 1-5: 5 comparisons for MRC-5; sheet 6-10: 5 comparisons for HFF (see Table 1). Each Sheet contains 15 columns and the list of DEG as rows. Only genes with FDR < 0.05 by DESeq and edgeR are listed.
Column 1-4: gene annotation
Column 5-10: RPKM values for each single sample
Column 11-13: mean RPKM values and log2 fold-change
Column 14-15: adjusted p-values (FDR) by DESeq and edgeR

Supplementary Table 2:
Excel table with fuzzy c-mean clustering results.
Column 1-4: gene annotation
Column 5-14: mean RPKM values of MRC-5 and HFF and all five PD
Column 15: cluster membership number [1-5]

Supplementary Table 3:
Excel table with functional enrichment analysis (FungiFun). The file contains 9 sheets: One sheet for each single cluster used for GO enrichment analysis; two sheets with GO results for combined clusters; and two sheets with KEGG results for combined clusters. Each sheet contais the pathway/term IDs, the corresponding names, toplevels, exact p-values, adjusted p-values, number of genes per pathway/term and per input list.

Supplementary Table 4:
Excel table with detailed results from the monotony test. Commonly significantly found genes are highlighted by red and green background color.
Column 1-3: gene annotation
Column 4-5: Spearman correlation values and p-values (cor.test) for MRC-5
Column 6-7: Spearman correlation values and p-values (cor.test) for HFF
